# Diabetes, hemoglobin A1c, and cognitive performance in older adults: is there any impact of frailty? Evidence from the ELSI-Brazil study

**DOI:** 10.1590/1414-431X2023e12939

**Published:** 2024-02-19

**Authors:** J.G. Souza, D.S. Farias-Itao, M.J.R. Aliberti, T.S. Alexandre, C. Szlejf, C.P. Ferri, M.F. Lima-Costa, C.K. Suemoto

**Affiliations:** 1 Laboratório de Investigação Médica no Envelhecimento (LIM-66), Serviço de Geriatria, Hospital das Clínicas, Faculdade de Medicina, Universidade de São Paulo, São Paulo, SP, Brasil; 2 Departamento de Patologia, Faculdade de Medicina, Universidade de São Paulo, São Paulo, SP, Brasil; 3 Instituto de Pesquisa, Hospital Sírio-Libanês, São Paulo, SP, Brasil; 4 Departamento de Gerontologia, Universidade Federal de São Carlos, São Carlos, SP, Brasil; 5 Hospital Israelita Albert Einstein, São Paulo, SP, Brasil; 6 Departamento de Psiquiatria, Universidade Federal de São Paulo, São Paulo, SP, Brasil; 7 Instituto de Pesquisa René Rachou, Fundação Oswaldo Cruz, Belo Horizonte, MG, Brasil

**Keywords:** Frailty, Epidemiology, Cognition, Diabetes complications, Diabetes mellitus

## Abstract

The aim of this study was to evaluate the association between diabetes and cognitive performance in a nationally representative study in Brazil. We also aimed to investigate the interaction between frailty and diabetes on cognitive performance. A cross-sectional analysis of the Brazilian Longitudinal Study of Aging (ELSI-Brazil) baseline data that included adults aged 50 years and older was conducted. Linear regression models were used to study the association between diabetes and cognitive performance. A total of 8,149 participants were included, and a subgroup analysis was performed in 1,768 with hemoglobin A1c data. Diabetes and hemoglobin A1c levels were not associated with cognitive performance. Interaction of hemoglobin A1c levels with frailty status was found on global cognitive z-score (P-value for interaction=0.038). These results suggested an association between higher hemoglobin A1c levels and lower cognitive performance only in non-frail participants. Additionally, undiagnosed diabetes with higher hemoglobin A1c levels was associated with both poor global cognitive (β=-0.36; 95%CI: -0.62; -0.10, P=0.008) and semantic verbal fluency performance (β=-0.47; 95%CI: -0.73; -0.21, P=0.001). In conclusion, higher hemoglobin A1c levels were associated with lower cognitive performance among non-frail participants. Higher hemoglobin A1c levels without a previous diagnosis of diabetes were also related to poor cognitive performance. Future longitudinal analyses of the ELSI-Brazil study will provide further information on the role of frailty in the association of diabetes and glycemic control with cognitive decline.

## Introduction

Currently, approximately 50 million adults have dementia worldwide (1). According to the 2020 Lancet Commission report, diabetes is a modifiable risk factor for dementia in later life (2). Individuals with diabetes have heterogeneous health conditions, varying from healthy individuals to those with impaired mental and functional capacities who have higher morbidity and lower life expectancy. Better management of diabetes in older adults depends on differentiating these distinct profiles of individuals according to the presence of age-related conditions that impact functionality (i.e. physical disability, sensory deficits, and cognitive impairment) (3).

Recent studies have shown an association between diabetes and cognitive performance in high-income and low-to-middle-income countries (4-10). For example, the presence of diabetes led to a 27% higher risk of dementia and cognitive impairment in older patients in the Health and Retirement Study (HRS), a nationally representative sample of older Americans (6). Diabetes was also a risk factor for dementia in the Mexican Health and Aging Study (MHAS) (7). In the Brazilian Longitudinal Study of Aging (ELSI-Brazil), participants with diabetes had 49% greater odds of having impaired memory than those without diabetes. However, when combining participants with undiagnosed and diagnosed diabetes, the association between diabetes and impaired memory was attenuated (8).

Frailty is a biological syndrome of decreased reserve and resistance to stressors, resulting from a cumulative decline of multiple physiological systems increasing vulnerability to adverse outcomes (11). This syndrome is related to a higher prevalence of diabetes and cognitive impairment (12-14). Moreover, a previous clinical trial demonstrated that frailty was an independent predictor of all outcomes in individuals with type 2 diabetes, including higher incidence of macro- and microvascular events, all-cause mortality, and death from cardiovascular causes (15).

While frailty represents an additional risk for older individuals with diabetes, interactions between these two conditions have been scarcely investigated. For example, the effect of intensive glucose treatment on decreasing macro- and microvascular events seems to be attenuated in frail adults (15). However, the role of frailty as an effect modifier in the association between diabetes and cognitive function remains unknown.

In this study, we aimed to investigate the associations of diabetes and glycemic control with cognitive performance in a nationally representative sample of people aged 50 years and older living in Brazil. Additionally, we examined whether frailty modifies the association of diabetes with cognitive performance.

## Material and Methods

### Participants

This is a cross-sectional analysis of baseline assessment data of the ELSI-Brazil study, which was conducted between 2015 and 2016. The ELSI-Brazil study is a nationally representative study of Brazilians aged 50 years and older. This study was conducted in 70 municipalities. Trained professionals visited the sampled households and enrolled 9,412 participants. The interviewers collected information on sociodemographic variables, lifestyle factors, medication, and comorbidities. They also performed physical tests, including anthropometric parameters (weight and height), muscle strength, gait speed, and cognitive evaluation. Blood sample analyses were conducted in a probabilistic subsample of 2,004 participants, including measures of glycated hemoglobin (HbA1c). Further details on the study design and data collection are available elsewhere (16). For the current study, we excluded participants with missing data for study variables and participants with a previous diagnosis of Alzheimer's disease (AD). The ethics committee of the Faculty of Medicine, University of São Paulo approved the study. The ELSI-Brazil study was also approved by the National Research Ethics Commission (CONEP) committee. All individuals provided informed consent before participation.

### Cognitive assessment

The cognitive assessment was similar to that of the HRS neuropsychological battery and included the following neuropsychological tests: 1) Immediate recall test, which consists of recalling a list of 10 words immediately after hearing; 2) Delayed recall test was conducted five minutes after applying other distracting tests. In both tests each correct answer was given 1 point up to a maximum of 10 points; 3) Temporal orientation was assessed by asking the participant the current date (day, month, and year) and weekday. Each correct answer was given 1 point up to a maximum of 4 points; 4) Semantic verbal fluency test (animal category), which consists of asking participants to recall the names of animals in one minute, with each animal scoring 1 point (17). The cognitive scores were converted to z-scores by subtracting each score from the sample mean and dividing by the sample standard deviation (SD). For the memory score, we calculated the z-scores of the immediate and delayed 10-word recall tests and then standardized the mean of the two tests. We also calculated the mean of all cognitive tests (memory, temporal orientation, and semantic verbal fluency z-scores). Then, we standardized the mean to obtain a global composite cognitive z-score.

### Diabetes evaluation

Diabetes was determined in participants who answered “yes” to the question: “Has a doctor ever told you that you have diabetes or high blood sugar?” This approach is frequently used in large population-based cohort studies and proved to capture mortality risk in older adults in a previous work (18). Diabetes duration was also categorized as <10 years or ≥10 years (19). In the representative subsample of individuals with blood tests available, we obtained data on HbA1C (high-performance LC: Premier Hb9210™ HbA1c - Trinity Biotech Plc., Ireland), and participants were classified into three HbA1C levels: 1) Normal: HbA1C <5.7%; 2) Pre-diabetes: HbA1C ≥5.7%; and <6.5%; 3) High: HbA1C ≥6.5% (20).

### Sociodemographic, lifestyle, and clinical variables

We selected sociodemographic characteristics such as age, sex, self-reported race (White, Black or Brown, and Other), and educational levels (0; 1 to 4, 5 to 8, and more than 8 years of formal education). Smoking status was defined as current, previous, or never smoker. Alcohol consumption was defined as a self-reported alcohol consumption in the last month of 5 or more drinks for men and 4 or more drinks for women on a single occasion (21). The presence of chronic health conditions was evaluated by asking participants if a physician had ever told them that they had the following diseases: dyslipidemia, hypertension, myocardial infarction, heart failure, and stroke. Weight and height were measured to calculate the body mass index (BMI). Obesity was considered as BMI values ≥30 kg/m^2^ (22).

### Frailty measurement

Frailty was assessed considering the five components of the physical frailty phenotype: weight loss, weakness, slow gait, exhaustion, and low physical activity (11,23). Participants with three or more of these characteristics were considered frail. This definition of frailty is the most used in studies worldwide, which facilitates comparison with other surveys (24). We used the following definitions for the five frailty components: 1) Self-reported unintentional weight loss: >3 kg in the last 3 months or BMI <18.5 kg/m^2^ (23); 2) Weakness: assessed through handgrip strength using a dynamometer on the dominant upper limb. Each participant was instructed to apply the greatest possible strength in three attempts and the best performance was considered. For the definition of weakness, we used the sex- and BMI-specific lowest quintile as the cutoff point (Supplementary Table S1) (23); 3) Slow gait speed: defined by the time in seconds taken to walk three meters at the usual pace. We considered the fastest measures between two evaluations. Slow walking speed was defined using the sex- and height-specific highest quintile of time as the reference (Supplementary Table S2) (23); 4) Exhaustion: evaluated using the following questions from the Center for Epidemiologic Studies Depression Scale (CES-D) questionnaire: “In the last week, how often did you feel that you would not be able to carry on with your things (you started something, but you could not finish it)?” and “In the last week, how often did your routine activities require more effort to be completed?” Exhaustion was identified by a frequency greater than 3 to 4 times a day (25) and; 5) Physical inactivity: calculated using metabolic equivalents per week in kcal, based on the Short Form of the International Physical Activity Questionnaire (IPAQ). Participants in the sex-specific lowest quintile of kilocalories per week were considered to have physical inactivity (26).

### Laboratory assessment

In the representative subsample of participants with available blood tests, we also considered laboratory abnormalities that could interfere with the HbA1C interpretation. The following exams and parameters were considered: thyroid stimulating hormone levels <0.1 or >10 mU/L (chemiluminescence immunoassay: Advia Centaur XP, Siemens, Germany), hemoglobin levels <11 g/dL in men and <10 g/dL in women (automate Coulter^®^ LH 750 Hematology Analyzer, Beckman Coulter, USA); and serum creatinine levels >1.2 mg/dL (colorimetric, Jaffe reaction without deproteination: Advia 2400, Siemens) (27).

### Statistical analysis

For descriptive analysis, individuals with and without diabetes were compared using *t*-tests for continuous variables and chi-squared tests for categorical variables. To compare the three groups of Hba1C, we used one way-ANOVA for continuous variables and chi-squared tests for categorical variables.

Nested linear regression models were fitted to estimate associations of diabetes with cognitive performance. The dependent variables were composite global cognition, temporal orientation, memory, and semantic verbal fluency z-scores. The first adjusted model included sociodemographic variables (age, sex, race, and education). Lifestyle and clinical conditions were added to the second model (smoking, binge drinking, chronic diseases, diabetes duration, and frailty). To test whether frailty was an effect modifier for the associations of diabetes with performance in each cognitive test, an interaction term between diabetes and frailty was used in the fully adjusted models.

In the subgroup analysis comprising the representative subsample with available laboratory tests, linear regression models were fitted to examine associations of HbA1C levels with cognitive performance. The models were adjusted for sociodemographic factors, lifestyle, clinical variables, and laboratory abnormalities that could interfere with the HbA1C interpretation. We also tested whether frailty was an effect modifier for the associations of HbA1C levels with each cognitive performance test.

Finally, we investigated associations of diabetes status with cognitive performance. The independent variable was the combination of self-reported diabetes diagnosis and HbA1C measurements. Thus, the participants were divided into four groups: 1) No reported diabetes and HbA1C <8.0%; 2) No reported diabetes and HbA1C ≥8.0%; 3) Reported diabetes and HbA1C <8.0%; and 4) Reported diabetes and HbA1C ≥8.0% (28). The same linear regression models described above were fitted to the data.

The analyses were performed considering two-tailed tests and a significance level of 5%. We used sampling weights provided by the ELSI-Brazil study to account for the unequal probability of participant selection and complex survey design (16). Variance inflation factors (VIFs) were used to determine multicollinearity in all models. Analyses were performed using STATA/SE 17.0 software (StataCorp, USA).

## Results

**Figure 1:**
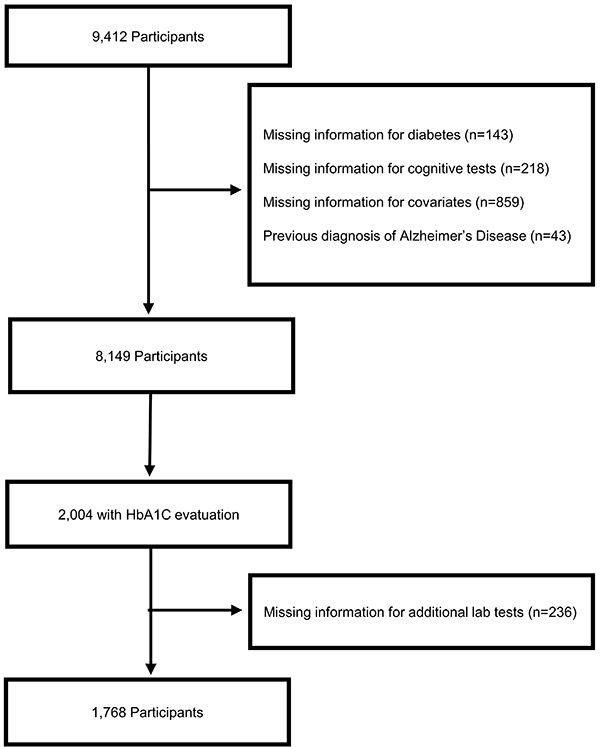
Flowchart of inclusion of study participants.

From a total sample of 9,412 participants, we excluded 1,220 individuals with missing information on diabetes, cognitive tests, and covariates. We also excluded 43 people with a previous diagnosis of AD (Figure 1). The final sample consisted of 8,149 participants. Participants had a mean age (SD) of 63 (9.5) years, 56% were women, 57% were Black or Brown. Regarding education, 14% had no formal education, 38% had 1 to 4 years, 21% had 5 to 8 years, and 27% had more than 8 years of formal education. Previous diagnosis of diabetes was reported by 15% of participants, and among them 37% had diabetes for 10 years or more. The presence of diabetes was associated with older age, lower frequency of current smoking and alcohol consumption, higher frequency of chronic health conditions, and lower scores in the immediate and delayed word recall test. Diabetes was also associated with a higher prevalence of frailty (Table 1).

**Table 1:** Participant characteristics according to self-reported diabetes (n=8,149)

Variables	Diabetes(n=1,231)	Without Diabetes(n=6,918)	P-value
Age (years), mean (SD)*	64.2 (9.2)	62.4 (9.5)	<0.001
Women (%)^#^	57.9	55.4	0.098
Education (years) (%)^#^			0.116
0	15.2	14.1	
1 to 4	40.2	37.1	
5 to 8	20.3	21.2	
>8	24.3	27.0	
Race/ethnicity (%)^#^			0.113
White	41.5	39.3	
Black or Brown	54.5	57.4	
Other	4.0	3.3	
Current smoker (%)^#^	13.2	17.8	<0.001
Binge drinking (%)^#^	7.5	9.8	0.010
Dyslipidemia (%)^#^	41.2	29.8	<0.001
Hypertension (%)^#^	66.2	50.0	<0.001
Obesity, (%)^#^	37.6	28.2	<0.001
Myocardial infarction (%)^#^	10.6	4.7	<0.001
Heart failure (%)^#^	10.9	6.4	<0.001
Stroke (%)^#^	6.9	3.9	<0.001
Frailty (%)^#^	15.4	9.0	<0.001
Cognitive performance			
Immediate word recall, mean (SD)*	4.2 (1.6)	4.3 (1.6)	<0.001
Delayed word recall, mean (SD)*	2.8 (1.8)	2.9 (1.9)	0.015
Semantic verbal fluency, mean (SD)*	11.9 (4.1)	12.1 (4.3)	0.075
Temporal orientation, mean (SD)*	3.5 (0.9)	3.5 (0.9)	0.635

SD: Standard deviation. *Independent-samples *t*-test for continuous variables; ^#^chi-squared test for categorical variables.

In the subgroup analysis with the 2,004 participants with HbA1C available, we excluded 236 individuals with missing values on other laboratory tests. Therefore, our final sample comprised 1,768 participants (Figure 1). Participants with HbA1C ≥6.5% were older, had lower educational levels, higher frequency of Black and Brown races, as well as a higher frequency of chronic diseases and frailty. This group also had a lower frequency of alcohol consumption and lower scores in the immediate word recall test. (Supplementary Table S3).

**Figure 2:**
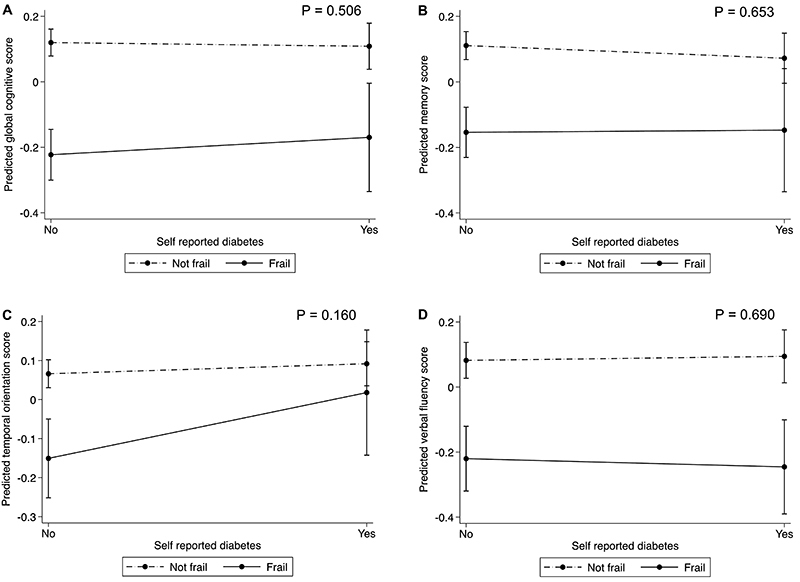
Frailty as an effect modifier of the association between self-reported diabetes and (**A**) global cognitive z-score; (**B**) memory z-score; (**C**) temporal orientation z-score; and (**D**) semantic verbal fluency z-score. Estimates were calculated using linear regression models adjusted for age, sex, race, education, dyslipidemia, hypertension, obesity, myocardial infarction, heart failure, stroke, smoking status, binge drinking, and diabetes duration.

**Figure 3:**
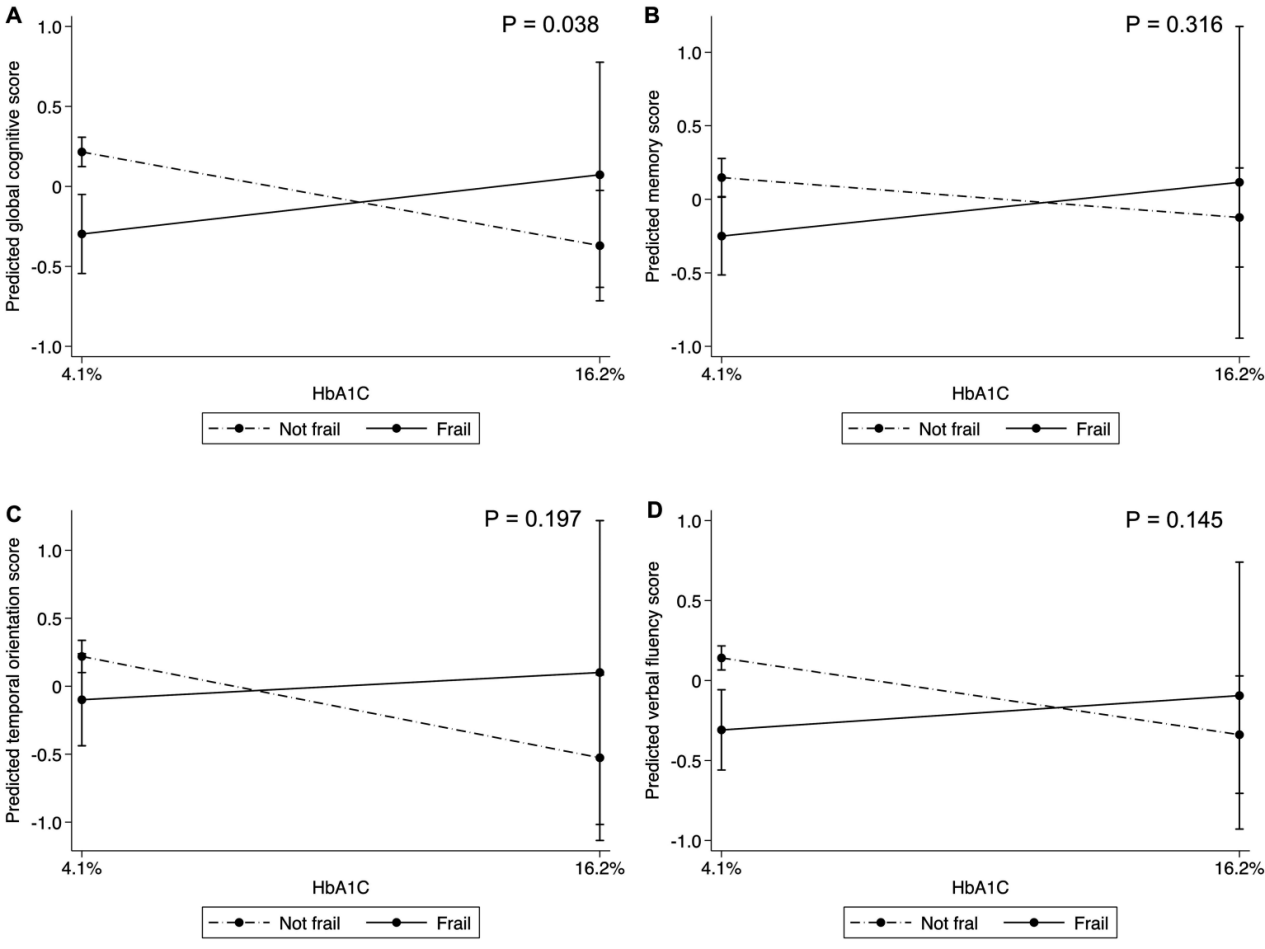
Frailty as an effect modifier of the association between glycated hemoglobin (HbA1c) and (**A**) global cognitive z-score, (**B**) memory z-score, (**C**) temporal orientation z-score, and (**D**) semantic verbal fluency z-score. Estimates were calculated using linear regression models adjusted for age, sex, race, education, dyslipidemia, hypertension, obesity, myocardial infarction, heart failure, stroke, smoking status, binge drinking, insulin, diabetes duration, and laboratory changes that can interfere with the glycated hemoglobin levels (thyroid stimulating hormone levels <0.1 or >10 mU/L, hemoglobin levels <11 g/dL in men and <10 g/dL in women, and serum creatinine levels >1.2 mg/dL).

In the models adjusted for sociodemographic, lifestyle, and clinical conditions, no association was found between diabetes and cognitive performance (Table 2), as well as between different HbA1C level groups and cognitive performance (Table 3). Multicollinearity was observed between insulin use and self-reported diabetes (variance inflation factor VIF >10). Therefore, insulin use was not included in the regression model. There were no interactions between diabetes and frailty for any cognitive outcomes (Figure 2). However, frailty status was an effect modifier in the association between HbA1C levels and global cognitive z-score (P-value for interaction= 0.038) (Figure 3). Higher hemoglobin A1c levels were associated with lower cognitive performance only among non-frail participants. Finally, regarding diabetes diagnosis and control, we found that people having undiagnosed diabetes with higher HbA1C levels were associated with lower composite global cognitive z-score and worse semantic verbal fluency performance (Table 4).

**Table 2:** Association of self-reported diabetes with cognitive performance (n=8,149).

	Unadjusted Model		Model 1		Model 2	
	β (95%CI)	P-value	β (95%CI)	P-value	β (95%CI)	P-value
Composite global score	-0.06 (-0.13; 0.00)	0.053	-0.01 (-0.07; 0.05)	0.818	-0.01 (-0.10; 0.08)	0.852
Temporal orientation	0.02 (-0.04; 0.09)	0.492	0.05 (-0.02; 0.11)	0.151	0.03 (-0.06; 0.12)	0.468
Memory	-0.10 (-0.18; -0.02)	0.013	-0.04 (-0.12; 0.04)	0.364	-0.03 (-0.14; 0.09)	0.654
Semantic verbal fluency	-0.03 (-0.11; 0.05)	0.435	0.00 (-0.06; 0.07)	0.909	-0.01 (-0.10; 0.08)	0.833

CI: confidence interval. Cognitive test scores were standardized (z-score). Model 1: Linear regression model adjusted for age, sex, race, and education. Model 2: Linear regression model adjusted for age, sex, race, education, dyslipidemia, hypertension, obesity, myocardial infarction, heart failure, stroke, smoking status, binge drinking, diabetes duration, and frailty.

**Table 3:** Association of glycated hemoglobin (HbA1) levels with cognitive performance (n=1,768).

	Unadjusted Model		Model 1		Model 2		Model 3	
	β (95%CI)	P-value	β (95%CI)	P-value	β (95%CI)	P-value	β (95%CI)	P-value
Composite global score								
HbA1C ≥5.7% and <6.5%	-0.03(-0.16; 0.10)	0.656	0.04(-0.05; 0.12)	0.409	0.02(-0.07; 0.12)	0.629	0.02(-0.07; 0.11)	0.652
HbA1C ≥6.5%	-0.22(-0.43; -0.02)	0.030	-0.01(-0.16; 0.13)	0.881	-0.05(-0.21; 0.11)	0.524	-0.05(-0.21; 0.11)	0.562
Temporal orientation								
HbA1C ≥5.7% and <6.5%	0.02(-0.09; 0.12)	0.768	0.04(-0.05; 0.14)	0.389	0.04(-0.06; 0.14)	0.409	0.04(-0.06; 0.13)	0.436
HbA1C ≥6.5%	-0.06(-0.23; 0.11)	0.474	0.01(-0.14; 0.17)	0.843	0.02(-0.13; 0.16)	0.844	0.02(-0.13; 0.17)	0.793
Memory								
HbA1C ≥5.7% and <6.5%	-0.01(-0.16; 0.14)	0.880	0.04(-0.05; 0.14)	0.367	0.03(-0.08; 0.14)	0.539	0.03(-0.08; 0.14)	0.543
HbA1C ≥6.5%	-0.20(-0.38; -0.03)	0.023	-0.01(-0.15; 0.14)	0.936	-0.03(-0.19; 0.13)	0.740	-0.03(-0.19; 0.14)	0.750
Semantic verbal fluency								
HbA1C ≥5.7% and <6.5%	-0.08(-0.20; 0.04)	0.186	-0.02(-0.12; 0.08)	0.697	-0.04(-0.14; 0.06)	0.469	-0.04(-0.14; 0.06)	0.447
HbA1C ≥6.5%	-0.22(-0.41; -0.03)	0.024	-0.04(-0.16; 0.08)	0.549	-0.11(-0.26; 0.04)	0.137	-0.11(-0.26; 0.04)	0.157

HbA1C: Glycated hemoglobin, CI: confidence interval. Cognitive test scores were standardized (z-score). Reference: Participants with HbA1C <5.7% (n=652). Model 1: Linear regression model adjusted for age, sex, race, and education. Model 2: Linear regression model adjusted for age, sex, race, education, dyslipidemia, hypertension, obesity, myocardial infarction, heart failure, stroke, smoking status, binge drinking, insulin use, diabetes duration, and frailty. Model 3: Linear regression model adjusted for age, sex, race, education, dyslipidemia, hypertension, obesity, myocardial infarction, heart failure, stroke, smoking status, binge drinking, insulin use, diabetes duration, frailty, and laboratory changes that can interfere with the glycated hemoglobin levels (thyroid stimulating hormone levels <0.1 or >10 mU/L, hemoglobin levels <11 g/dL in men and <10 g/dL in women, and serum creatinine levels >1.2 mg/dL).

**Table 4:** Association of self-reported diabetes and glycated hemoglobin levels with cognitive performance (n=1768).

	Without self-reported diabetes and HbA1C ≥8.0% (n=29)		Self-reported diabetes and HbA1C <8.0% (n=201)		Self-reported diabetes and HbA1C ≥8.0% (n=96)	
	β (95%CI)	P-value*	β (95%CI)	P-value*	β (95%CI)	P -value*
Composite global score	-0.36(-0.62; -0.10)	0.008	0.70(-2.06; 3.47)	0.616	0.76(-2.02; 3.55)	0.589
Temporal orientation	-0.14(-0.57; 0.28)	0.508	0.90(-2.39; 4.20)	0.589	0.80(-2.51; 4.10)	0.634
Memory	-0.24(-0.52; 0.05)	0.100	0.53(-2.64; 3.70)	0.741	0.63(-2.52; 3.78)	0.693
Semantic verbal fluency	-0.47(-0.73; -0.21)	<0.001	0.19(-1.83; 2.20)	0.856	0.28(-1.62; 2.18)	0.769

HbA1C: Glycated hemoglobin. CI: confidence interval. Cognitive test scores were standardized (z-score). Reference: Participants without self-reported diabetes and HbA1C <8.0% (n=1442). *Linear regression model adjusted for age, sex, race, education, dyslipidemia, hypertension, obesity, myocardial infarction, heart failure, stroke, smoking status, binge drinking, insulin use, diabetes duration, frailty, and laboratory changes that can interfere in the glycated hemoglobin levels (thyroid stimulating hormone levels <0.1 or >10 mU/L, hemoglobin levels <11 g/dL in men and <10 g/dL in women, and serum creatinine levels >1.2 mg/dL).

## Discussion

In this nationally representative sample of community-dwelling middle-aged and older adults from a low-to-middle-income country, diabetes and HbA1C levels were not associated with cognitive performance. Interestingly, an interaction of HbA1C levels with frailty status was found on cognitive performance. Higher HbA1C levels were associated with lower cognitive performance among non-frail participants. Additionally, higher HbA1C levels without a previous diagnosis of diabetes were also related to poor cognitive performance.

Compared to previous studies that found a strong relationship between diabetes and cognitive performance, our results showed an unexpected absence of association (4-10). Participants were older in the HRS than in the ELSI-Brazil study (mean age: 73 *vs* 63 years), which could explain a higher probability of detecting diabetes and cognitive impairment in the HRS since both conditions are associated with older age (6,29). Also, that study (6) showed that even though there was a higher proportion of dementia in participants with diabetes at baseline, individuals who developed incident diabetes during the follow-up did not have higher odds of dementia. This finding suggests that the associations with cognitive impairment may be weaker in participants with a shorter diabetes duration. In our study, most participants had a diabetes duration of less than 10 years, which may also explain the absence of association between diabetes and cognitive performance.

Only 15% of ELSI-Brazil participants reported having diabetes. Approximately 232 million (50%) people who have diabetes are unaware of their condition, and most non-diagnosed diabetes occurs in low-to-middle-income countries (29). Analyzing the MHAS sample, we observed a similar profile considering that among 5,398 Mexicans, 25% had no formal education and only 14% reported having diabetes. In the MHAS, participants with diabetes had a 2-fold higher probability of developing dementia after two years of follow-up (7). Compared to our findings, these differences could be explained by the longitudinal design of the Mexican study.

In a meta-analysis that included 14 longitudinal studies from different countries with 2,310,330 individuals, diabetes was associated with a 62% increased risk of dementia in both sexes. This analysis included heterogeneous studies with different socioeconomic profiles and age ranging from 43 to 83 years. Among them, the MHAS was the only Latin-American study. The authors concluded that the magnitude of the relationship in the association between diabetes and dementia differs according to characteristics of each population (4). In another review that studied risk factors for dementia, the associated risk of diabetes for dementia was larger in studies that had a long follow-up time (5). These findings suggest that future follow-up assessments of the ELSI-Brazil study will be important to explore other nuances of the associations between diabetes and cognition, including cognitive decline.

In addition, we did not find any association between different HbA1C level groups and cognitive outcomes. This finding differs from a previous ELSI-Brazil study that found that self-reported diabetes and HbA1c levels ≥6.5% were associated with memory impairment (8). On the other hand, we did not find an association between diabetes and memory after adjustment for sociodemographic variables. The previous ELSI-Brazil study differed from ours in some aspects. First, we used z-scores to approach cognitive performance. Furthermore, in the previous ELSI-Brazil study, the authors did not adjust the analysis for race when memory was the outcome and for race and education when verbal fluency was the outcome (2,8). Also, we analyzed data from a different sample since we excluded participants without information on race and frailty status.

An association was found between higher HbA1C levels and poor global cognitive performance and verbal fluency in participants with undiagnosed diabetes. Individuals who are unaware of the disease represent a group at a higher risk of dementia because they do not have access to interventions that can reduce complications (30). Our results reinforce the importance of patient involvement in diabetes treatment, including training in skills that are important for the prevention of organ damage (self-care, problem-solving, decision-making, and active collaboration). In addition, cognitively impaired individuals, especially those with low social support, may have greater difficulty in diabetes monitoring and control (31).

We considered frailty as a possible effect modifier in the association of diabetes or HbA1C levels with cognitive performance. Other authors have already demonstrated a higher frequency of diabetes in frail compared to non-frail older adults (13). Frailty is also associated with worse cognitive performance (14). In our study, we found a higher frequency of frailty in participants with diabetes. In addition, a trend towards a higher prevalence of frailty was also observed in participants with higher HbA1C values. Although we did not find interactions between diabetes and frailty for any cognitive outcomes, frailty status was an effect modifier of the association between HbA1C levels and cognitive performance. The lower prevalence of self-reported diabetes may be an explanation for the absence of interactions between diabetes and frailty in our study. Our results suggested an association between higher HbA1C levels and lower cognitive scores only in non-frail participants. These findings corroborate our initial hypothesis that frail individuals with lower blood glucose levels would have worse cognitive performance (32). In a previous ELSI-Brazil study, hypertension was also related to cognitive impairment only in non-frail older adults (33). These findings strengthen the need for an individualized approach to chronic disease control in frail individuals (28).

The link between diabetes and cognition could be explained by insulin resistance, hyperglycemic toxic effects (mediated by the polyol and hexosamine pathways), accumulation of advanced glycation products, and chronic inflammatory processes. All of these may affect brain tissue directly or lead to vascular changes and brain infarcts. These processes can also interfere with amyloid metabolism, increasing the incidence of AD (9). Individuals with diabetes usually have abnormalities in structural and functional neuroimaging exams, including cortical and subcortical atrophy, cerebral infarcts, and hypometabolism in typical regions of AD detected by positron emission tomography scan (34,35).

Our study had some limitations. The cross-sectional design does not allow establishing causal relationships between diabetes and cognitive function. Longitudinal data from ELSI-Brazil will be important to determine the association between diabetes and cognitive decline. In addition, while we excluded participants with a previous AD diagnosis, the ELSI-Brazil study did not collect data on other dementia types, which cannot be excluded from our sample. Another limitation is that a formal dementia evaluation was not performed. We investigated the association between diabetes and cognitive performance, which has a less clear clinical meaning than dementia diagnosis. Although the global cognitive assessment adopted in the ELSI-Brazil study is harmonized with other large-scale studies, such as the HRS, a formal validation of the cognitive battery was not performed in Brazil (17). However, the individual cognitive assessments, such as the 10-word recall test, animal verbal fluency, and temporal orientation assessment, have been widely used for cognitive assessment in Brazil and worldwide (36-40). Finally, the HbA1C evaluation was available for a smaller subgroup, which could have limited the possibility of finding other significant associations.

However, this study has some strengths considering that ELSI-Brazil is the first nationally representative population-based survey that allowed the analysis of epidemiological data in adults aged 50 years and older in Brazil. Contrary to previous studies on this topic, our sample was mainly non-white and with low education level. In addition, our study is one of the first that sought to investigate interactions between frailty and diabetes or HbA1C levels on cognitive performance.

In conclusion, diabetes and HbA1C levels were not related to cognitive performance. Higher HbA1C levels were associated with poor cognitive performance only in non-frail participants. Additionally, higher HbA1C levels without a previous diabetes diagnosis were related to poor cognitive performance. Future longitudinal analyses of the ELSI-Brazil study, as soon as the follow-up assessments become available, will provide further information on the role of frailty in cognitive decline in older people with diabetes. In addition, they will allow a greater understanding of the causal pathway of the associations of diabetes and glycemic control with cognitive decline.

## Supplementary Material

Click to view [pdf].
