# Supplementary material for: Diabetes, hemoglobin A1c, and cognitive performance in older adults: is there any impact of frailty? Evidence from the ELSI-Brazil study
Source: Braz J Med Biol Res. 2024 Feb 19;57:e12939. doi: 10.1590/1414-431X2023e12939 (PMC10880886; doi:10.1590/1414-431X2023e12939)
Supplement: Supplementary file 1 [file 1414-431X-bjmbr-57-e12939-suppl.pdf]

**Table S1.** Definition of weakness by the lowest quintile as the cutoff point, according to the sex and body mass index percentile groups.

| Men (n=3,605) | Women (n=4,544) |
|---|---|
| Hg ≤23 and BMI interval (12.9 to 23.9) | Hg ≤14 and BMI interval (11.8 to 24.6) |
| Hg ≤27 and BMI interval (24.0 to 26.6) | Hg ≤15 and BMI interval (24.7 to 28.0) |
| Hg ≤28 and BMI interval (26.7 to 29.7) | Hg ≤16 and BMI interval (28.1 to 31.8) |
| Hg ≤28 and BMI interval (29.8 to 50.0) | Hg ≤16 and BMI interval (31.9 to 58.0) |

Hg: Hand grip values measured in kilograms; BMI: Body mass index measured in weight divided by height squared; 25th percentiles values were used.

**Table S2.** Definition of low walking speed by the greatest quintile of time, stratified according to sex and height.

| Men (n=3,605) | Women (n=4,544) |
|---|---|
| Time ≥4.89 and height interval (1.35 to 1.66) | Time ≥5.62 and height interval (1.24 to 1.53) |
| Time ≥4.52 and height interval (1.67 to 1.95) | Time ≥5.23 and height interval (1.54 to 1.82) |

Height interval in meters. Median values were used. Time: time in seconds taken to walk three meters.

**Table S3.** Participant characteristics according to glycated hemoglobin levels (n=1,768).

| Variables | HbA1C <5.7% (Normal) (n=652) | HbA1C ≥5.7% and <6.5% (Prediabetes) (n=791) | HbA1C ≥6.5% (High) (n=325) | P-value |
|---|---|---|---|---|
| Age (years), mean (SD)* | 62.5 (9.0) | 63.6 (9.3) | 64.7 (9.1) | 0.001 |
| Women (%)# | 58.4 | 69.3 | 62.8 | <0.001 |
| Education (years) (%)# | | | | 0.019 |
| 0 | 9.7 | 12.8 | 12.9 | |
| 1 to 4 | 40.5 | 39.7 | 45.2 | |
| 5 to 8 | 21.3 | 17.8 | 21.2 | |
| >8 | 28.5 | 29.7 | 20.6 | |
| Race/ethnicity (%)# | | | | 0.005 |
| White | 43.6 | 38.8 | 31.4 | |
| Black or Brown | 52.4 | 58.0 | 64.9 | |
| Other | 4.0 | 3.2 | 3.7 | |
| Current smoker (%)# | 15.8 | 13.8 | 12.0 | 0.018 |
| Binge drinking, (%)# | 11.4 | 5.6 | 5.8 | <0.001 |
| Dyslipidemia (%)# | 31.9 | 40.2 | 45.8 | <0.001 |
| Hypertension (%)# | 51.1 | 55.4 | 65.2 | <0.001 |
| Obesity (%)# | 23.3 | 33.9 | 41.8 | <0.001 |
| Myocardial infarction (%)# | 2.9 | 6.3 | 7.4 | 0.003 |
| Heart failure (%)# | 7.8 | 6.1 | 6.8 | 0.422 |
| Stroke (%)# | 4.6 | 4.7 | 7.1 | 0.196 |
| Frailty (%)# | 7.3 | 8.8 | 15.3 | 0.005 |
| Cognitive Performance* | | | | |
| Immediate word recall (0–10), mean (SD) | 4.4 (1.5) | 4.3 (1.6) | 4.14 (1.6) | 0.045 |
| Delayed word recall (0–10), mean (SD) | 2.9 (1.8) | 3.0 (1.9) | 2.70 (1.9) | 0.053 |
| Semantic verbal fluency, mean (SD) | 12.2 (4.3) | 11.9 (4.4) | 11.60 (4.3) | 0.130 |
| Temporal orientation (0–4), mean (SD) | 3.6 (0.8) | 3.6 (0.8) | 3.50 (1.0) | 0.360 |

HbA1C: glycated hemoglobin; SD: standard deviation. *ANOVA test for continuous variables; #chi-squared test for categorical variables.
